# Supplementary material for: Transcriptome Analysis in Mexican Adults with Acute Lymphoblastic Leukemia
Source: Int J Mol Sci. 2024 Feb 1;25(3):1750. doi: 10.3390/ijms25031750 (PMC10855968; doi:10.3390/ijms25031750)
Supplement: Supplementary file 1 [file ijms-25-01750-s001.zip › Supplementary figures.pdf]

International Journal of
Molecular Sciences

MDPI

Supplementary Material

1. Supplementary Data

1.1 Supplementary tables

Supplemental Table S1. Sequences of the primers used in validation of Microarray results through Real-Time PCR.
Supplemental Table S2. Top molecules with the Highest Node Degree in the Interaction Network.
Supplemental Table S3. Biological processes enriched in KEEG analysis using STRING nodes.
Supplemental Table S4. Gene correlation coefficients in the main correlation module**.**
Supplemental Table S5. Potential Therapeutic Target Genes in Acute Lymphoblastic Leukemia.

1.2 Supplementary Figures

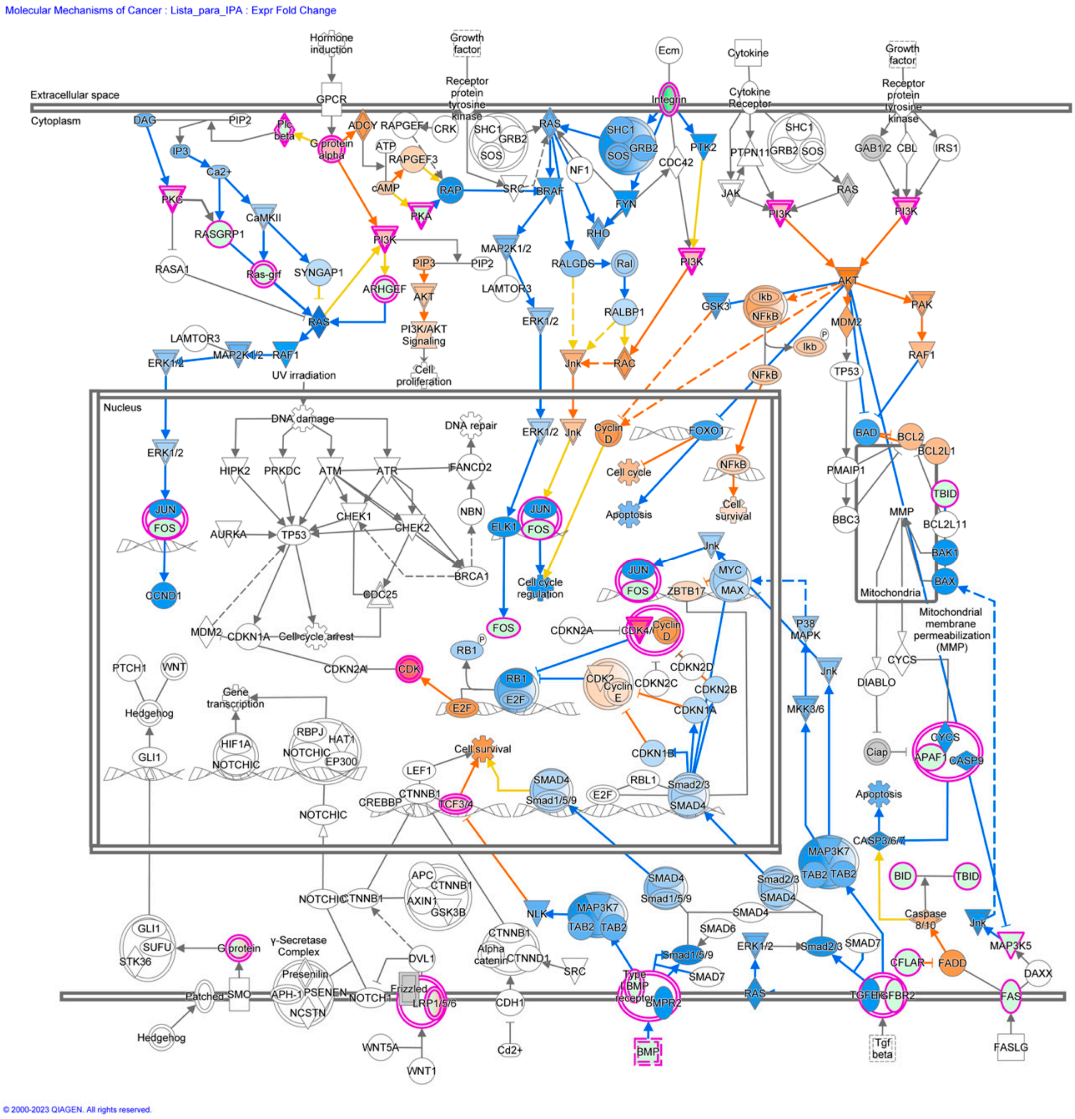

Supplemental Figure S1. Molecular mechanism of cancer in which differential expressed genes in ALL of adults in contrast to healthy subjects. Figure was generated by IPA (QIAGEN IPA; http://www.ingenuity.com).

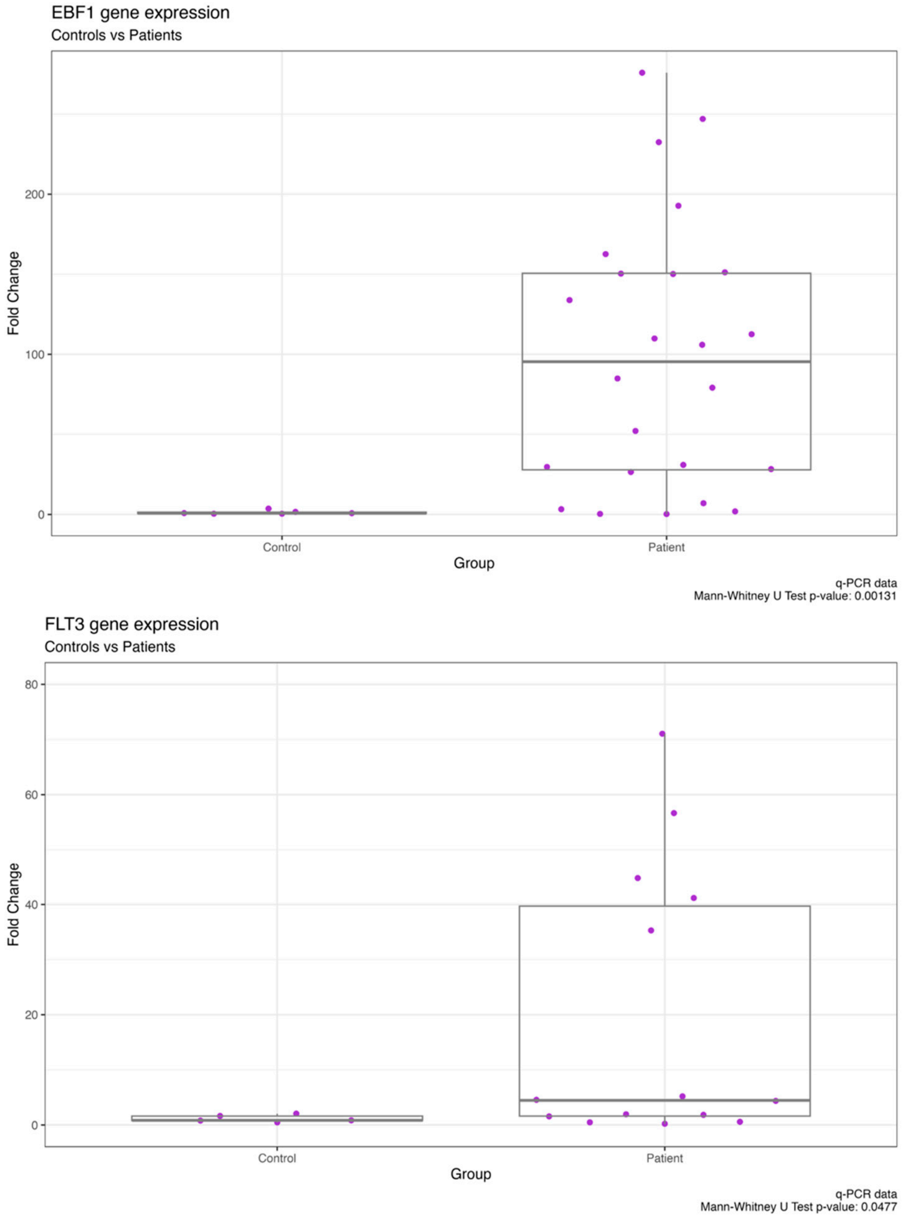

Supplemental Figure S2. Validation of Microarray results using Real-Time PCR for Key Genes. Validation of the differential expression analysis results for the genes *EBF1* and *FLT*3 using double delta Ct method **in a subset of patients**. *EBF1* and *FLT*3 were up-regulated in patients than controls. The statistical significance of the results is demonstrated through Mann-Whitney U tests ($p < 0.05$).
